# Electrochemical determination of L-tryptophan in food samples on graphite electrode prepared from waste batteries

**DOI:** 10.1038/s41598-022-09472-7

**Published:** 2022-03-31

**Authors:** Žaklina Z. Tasić, Marija B. Petrović Mihajlović, Milan B. Radovanović, Ana T. Simonović, Dragana V. Medić, Milan M. Antonijević

**Affiliations:** Technical Faculty in Bor, University of Belgrade, VJ 12, P.O. Box 50, 19210 Bor, Serbia

**Keywords:** Sensors and biosensors, Electrochemistry

## Abstract

One of the goals of this research was to develop an electrochemical sensor that had the ability to determine the target analyte and was both cheap and non-toxic. Another goal was to influence the reduction of electronic waste. In accordance with these, a graphite rod from zinc-carbon batteries was used to prepare an electrochemical sensor for the determination of L-tryptophan in Britton–Robinson buffer solution. Two electrochemical methods were used in the experimental research, differential pulse voltammetry and cyclic voltammetry. The effect of different parameters, including the pH value of supporting solution, scan rate, as well as the concentration of L-tryptophan on the current response, was studied. The pH value of Britton–Robinson buffer influenced the intensity of L-tryptophan oxidation peak, as well as the peak potential. The intensity of the current response was the highest at pH 4.0, while the peak potential value became lower as the pH increased, indicating that protons also participated in the redox reaction. Based on the obtained data, electrochemical oxidation of L-tryptophan at the graphite electrode was irreversible, two electron/two proton reaction. In addition, it was observed that the oxidation peak increased as the scan rate increased. According to the obtained electrochemical data, it was suggested that the oxidation of L-tryptophan was mixed controlled by adsorption and diffusion. The linear correlation between oxidation peak and L-tryptophan concentration was investigated in the range 5.0–150.0 µM and the obtained values of limit of detection and limit of quantification were 1.73 µM and 5.78 µM, respectively. Also, the prepared electrochemical sensor was successful in determination of target analyte in milk and apple juice samples.

## Introduction

L-tryptophan (TRP) belongs to the essential amino acids because human body does not have the ability to synthesize it^1^. L-tryptophan is of multiple significance for humans. It is an important ingredient in the diet and is mainly found in foods rich in proteins such as dairy products, meat, seafood, soy, or nuts^2^. In addition to being an essential component of protein, L-tryptophan also participates in the synthesis of niacin, which is a precursor of important biomolecules in the body such as melatonin and serotonin^3^. Knowing L-tryptophan levels is very important because its deficiency can lead to metabolic and neurological disorders^4^. Having this in mind, the importance of determining this amino acid in biological samples, as well as in food samples can be accomplished. Although a number of classical methods^5–7^ are available to quantify this target analyte, electrochemical techniques have become important in this research field. Voltammetry is an electrochemical and electroanalytical technique based on measuring current as a function of applied potential. There are different types of voltammetric techniques, including polarography, cyclic voltammetry, and pulse voltammetric techniques (normal pulse, differential pulse, and square wave voltammetry)^8^. The advantages of voltammetric techniques are good sensitivity and a wide linear range of concentrations for both organic and inorganic analytes, short time needed for the analysis, wide choice of solvents and electrolytes that can be used in measurements, and the possibility of simultaneous determination of several different analytes without the need for their prior separation^9^. Cyclic voltammetric (CV) measurements are usually the first step during electrochemical studies of a compound, biological material, or electrode surface. The effectiveness of CV is reflected in the ability to quickly obtain information about the redox behavior of the target analytes in a wide range of potentials, thermodynamics of redox processes, kinetics of heterogeneous reactions, coupled chemical reactions, or adsorption processes^10^. CV is based on a linear change of the working electrode potential from the initial potential value to a predefined value, and then the potential changes at the same scan rate in the opposite direction to the initial or some other predetermined value^11^. Differential pulse voltammetry (DPV) has proven to be a very useful technique for determining traces of both organic and inorganic compounds. The application of potential pulse to electrodes leads to, in most experiments, a significant improvement in the ratio of Faraday and non-Faraday currents, because the Faraday current usually decreases more slowly over time compared to non-Faraday current (electric double layer charging current), which allows for the achievement of lower detection limits^12^. The difference between values of these currents is registered as a function of the applied potential resulting in the corresponding peak on the voltammogram whose height is directly proportional to the concentration of the measured analyte^13^. Moreover, the trend of increasing number of published review and research papers on the topic of electrochemical sensors indicates the importance of this field among researchers^14–17^. Zhao et al.^18^ used boron-doped diamond electrode as an electrochemical sensor for the detection of TRP in Na_2_PO_4_/NaOH buffer solution. Liu et al.^19^ used a silver-doped TiO_2_ nanoparticle modified glass carbon electrode to determine the TRP in 0.1 M KOH and 0.1 M phosphate buffer solutions. For the simultaneous detection of dopamine, uric acid, L-tryptophan and theophylline, a glassy carbon electrode modified with carbon dots (CDs/GCE) was used^20^. Carbon electrodes are widely used as sensors due to their good electrochemical properties such as low background current and good electrical conductivity. Additionally, they are relatively cheap, easy to prepare and mostly non-toxic. However, some researchers^4,21,22^ have suggested using a graphite rod from batteries instead of commercial electrodes since the development of technology also contributes to the generation of large amounts of waste. Zinc-carbon batteries can be found among such wastes. Improper disposal of waste batteries enables the release of heavy metals into the environment which can lead to numerous adverse effects on the living organisms^23,24^. Battery recycling would protect the environment and significant economic benefits could be achieved^25^. Due to good electrical conductivity and high surface area of graphite rod, it can be utilized as potential electrochemical sensor^4^. Additionally, the graphite rod is suitable for modification, which allows the development of sensors with better characteristics. According to the previous research papers^21,22^, the graphite rod is used as an electrochemical sensor for detection of myricetin antioxidants and tanninic acid.

The reuse of graphite from batteries to prepare a simple electrochemical sensor will also have an impact on the preservation of the environment In comparison to other research dealing with similar topics this paper presents the possibility of using waste graphite rod to prepare an electrochemical sensor. Thus, this is the one of possibilities of influencing the reduction of electronic waste collection. On the other hand, an electrochemical sensor can be obtained in a very simple way, which significantly reduces the cost of the process compared to other analytical methods. Additionally, graphite is a material that can be easily modified, and the characteristics of this prepared sensor can be improved with modifiers.

## Results and discussion

### Electrochemical behavior of L-tryptophan

**Figure 1:**
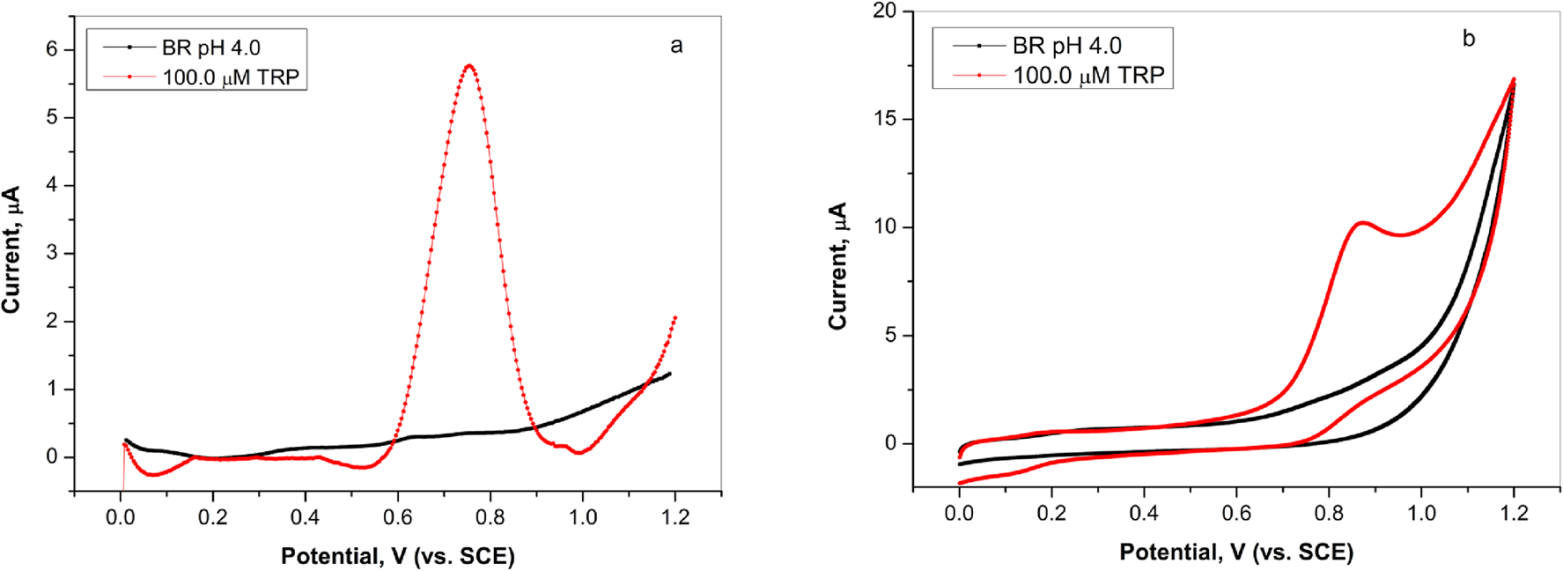
(**a**) Differential pulse voltammetry (curves subjected to baseline correction) and (**b**) cyclic voltammetry curves on graphite electrode in Britton–Robinson buffer solution (pH 4.0) in the absence and in the presence of 100.0 µM of L-tryptophan, scan rate 50.0 mV/s.

In order to determine the electrochemical behavior of L-tryptophan on a graphite electrode, differential pulse voltammetry measurements were performed. Figure 1a shows curves obtained in Britton–Robinson (BR) buffer (pH 4) without and with the addition of 100.0 µM TRP on graphite surface. The appearance of L-tryptophan oxidation peak at a potential of about 0.75 V (vs. SCE), compared to the curve recorded in the supporting solution, indicated that the graphite electrode had sensitivity characteristics. Furthermore, from cyclic voltammogram shown in Fig. 1b, it can be seen that L-tryptophan oxidation was an irreversible process which was in agreement with results presented in the literature^26^.

### Effect of solution pH

**Figure 2:**
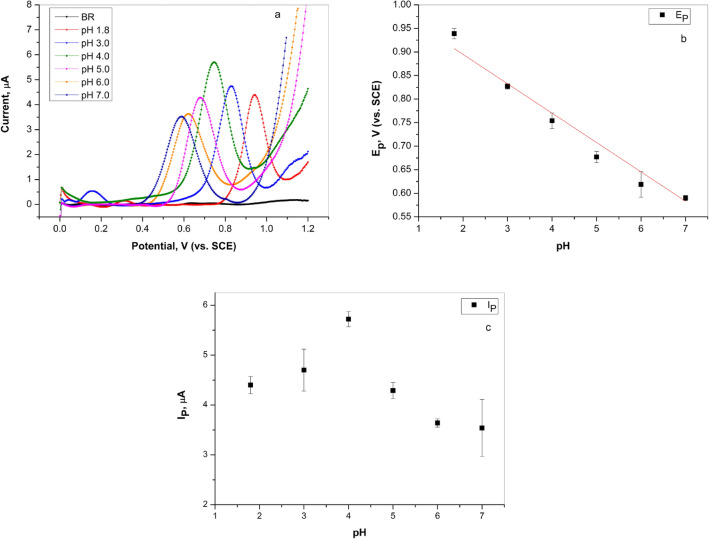
(**a**) Differential pulse voltammetry curves of 100.0 µM L-tryptophan on graphite electrode in Britton–Robinson buffer solution at different pH values, scan rate 50.0 mV/s (curves subjected to baseline correction); (**b**) Dependence of the peak potential of 100.0 µM L-tryptophan on pH value of the Britton–Robinson buffer solution; (**c**) Dependence of current peak of 100.0 µM L-tryptophan on pH value of the Britton–Robinson buffer solution.

In order to determine the optimal conditions for further research, the influence of the pH value of the Britton–Robinson solution (in range of 1.8–7.0) on the intensity of the oxidation peak of L-tryptophan (100.0 µM TRP) was examined. The recorded differential pulse voltammograms are illustrated in Fig. 2a. Since the highest current peak intensity was observed at pH 4.0, this solution was used for further analyses. In addition to the current peak intensity, the pH value of BR solution also affected the oxidation peak potential of L-tryptophan. According to the plot E_p(Trp)_–pH (Fig. 2b), the peak potential was shifted toward lower values as the pH increased indicating that protons also participated in the redox reaction. The linear relationship between E_p(Trp)_ and pH (Fig. 2b), can be expressed by Eq. (1). According to the Eq. (1), the obtained value of the slope was 62 mV/pH, which was approximate to the theoretical value of 59 mV/pH for a two-electron/two-proton process. Based on that, it can be assumed that an equal number of protons and electrons participated in the process at the electrode^27^. The intensity of current peak also changed with the pH (Fig. 2c). Increasing the pH value from 1.8 to 4 showed an increase in the value of the oxidation current peak. The highest value was recorded at pH 4. However, a further increase in the pH value of supporting electrolyte led to a decrease in the intensity of the current peak. This behavior might imply a decrease in the concentration of the protonated form of L-tryptophan^28^.

1 $$E_{p} = - \left( {\frac{0.0591m}{n}} \right)pH + b$$

2 $$E_{p} = 1.0194 - 0.0623\;{\text{pH}}, \;(R^{2} = 0.9821)$$

According to Nernst's Eq. (1)^3^ and the Eq. (2) the m/n ratio was calculated, as the ratio of the number of protons and electrons, which was 1.05 in this investigation . According to the literature^3^, if the m/n ratio is close to 1, it suggests that the same numbers of protons and electrons are involved in the reaction. Based on the obtained value of 1.05, and the previously mentioned assumptions, it can be said that the same number of protons and electrons participated in the electrochemical reaction of L-tryptophan on graphite electrode^3^.

### Oxidation mechanism of L-tryptophan

**Figure 3:**
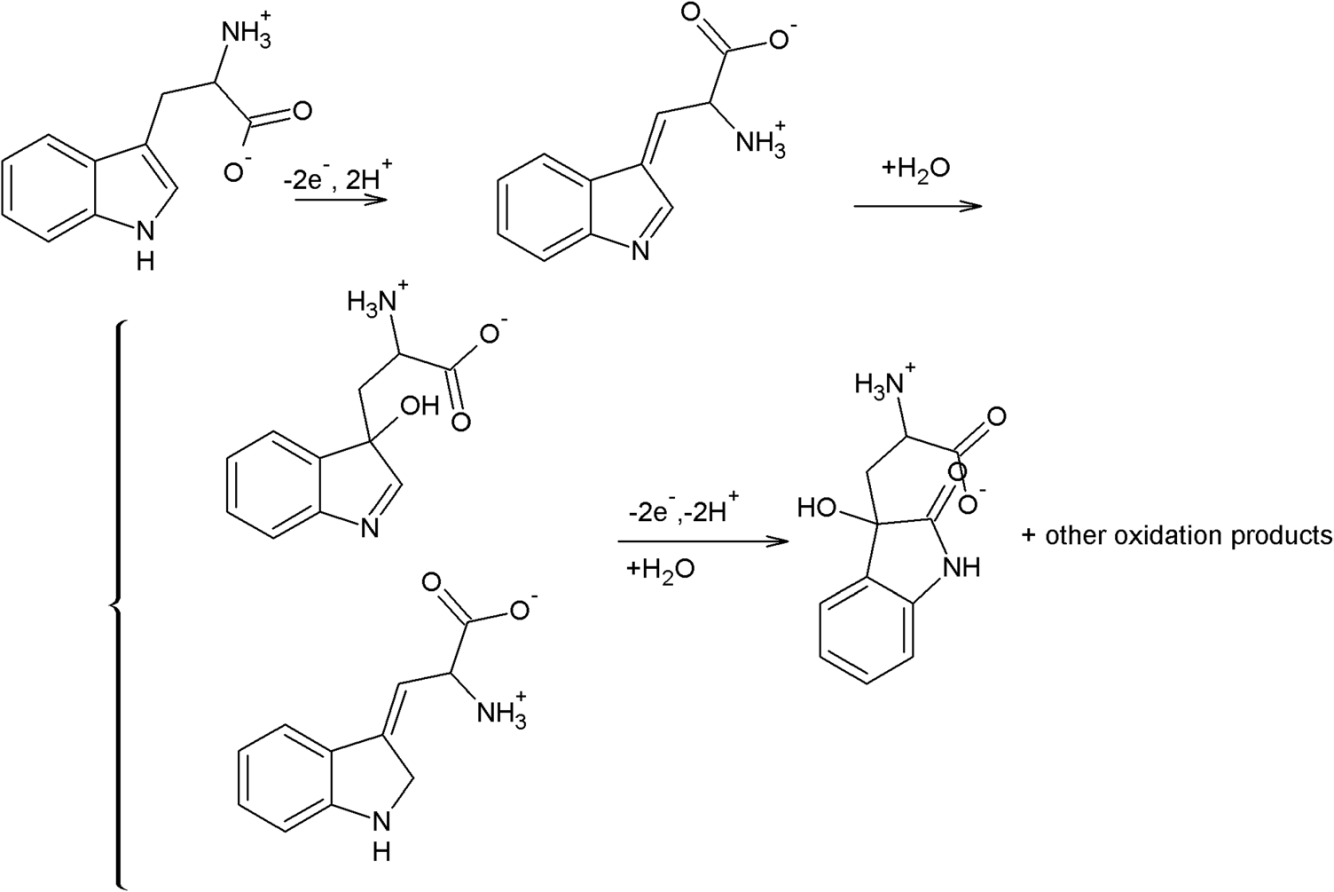
Mechanism of L-tryptophan oxidation.

According to the literature^29^, the oxidation mechanism of amino acids including L-tryptophan on the electrode surface has been described as an irreversible and multistage reaction. The achieved value of the m/n ratio (1.05) implied the participation of the same numbers of protons and electrons in the oxidation mechanism of L-tryptophan. In addition, a slope of 62 mV per pH unit was close to the theoretical value of 59 mV per pH, indicating a two-electron/two proton reaction. The adsorption of L-tryptophan on the graphite electrode occurred through a carboxyl group that facilitated electron transfer between the electrode and the indole as the electroactive part of the amino acid^29^. It can be assumed that L-tryptophan oxidized to 2-amino-3- (5-oxo-3,5-dihydro-2H-indol-3-yl) propionic acid (Fig. 3), which was indicated by the appearance of one irreversible oxidation peak on the cyclic voltammogram (Fig. 1b)^30^. Having in mind the literature data^3,26,30^ and the results obtained in this study, the mechanism of L-tryptophan oxidation on the graphite electrode was proposed (Fig. 3).

### Effect of scan rate

**Figure 4:**
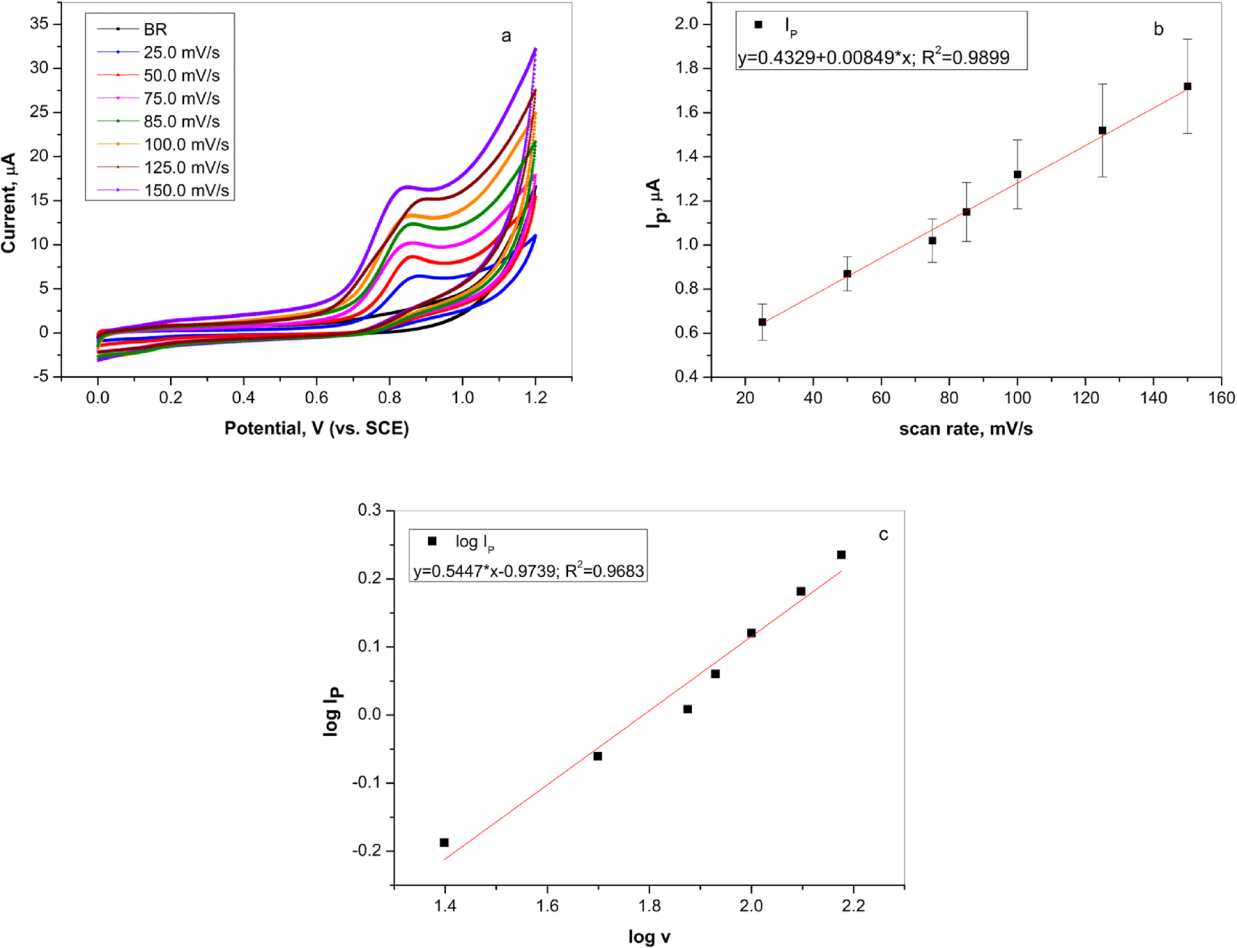
(**a**) Cyclic voltammetry curves of 100.0 µM L-tryptophan on graphite electrode in Britton–Robinson buffer solution (pH 4.0) at different scan rates (from 25.0 to 150.0 mV/s); (**b**) Dependence of peak current of 100.0 µM L-tryptophan on the scan rate; (**c**) Dependence of logarithm of peak current of 100.0 µM L-tryptophan on logarithm of scan rate.

The kinetics of the electrode reaction of L-tryptophan can be controlled by diffusion or adsorption and in accordance with that, the effect of scan rate on the current peak of L-tryptophan was examined by cyclic voltammetry. Figure 4a illustrates the recorded CV curves of L-tryptophan (100.0 µM) in Britton–Robinson solution (pH 4.0) with varied scan rates from 25.0 to 150.0 mV/s. According to the Fig. 4a, the oxidation peak increased as the scan rate increased. Further, by comparing the graphs Ip–v and logIp–logv (Fig. 4b, c) and the obtained regression coefficients (Eqs. 3 and 4), the better agreement of the current response of L-tryptophan was achieved with the scan rate, which was the characteristic of the adsorption-controlled process^3^. However, according to the logIp–logv graph and the corresponding Eq. (4), the slope value was 0.545. Thus, the achieved results suggest that the oxidation of TRP was mixed controlled by adsorption and diffusion, which was in accordance with the results obtained on the graphite pencil electrode^26^. A small difference in the value of the L-tryptophan peak potential with a change in scan rate was assumed to be because of the rapid redox reaction on graphite electrode^3,28^.

3 $$I_{p} (\upmu {\text{A}}) = 0.4329 + 0.00849v\;({\text{mV/s}})\;(R^{2} = 0.9899)$$

4 $$logI_{p} = 0.5447logv - 0.9739 \;(R^{2} = 0.9683)$$

### Determination of L-tryptophan on graphite electrode by differential pulse voltammetry

**Figure 5:**
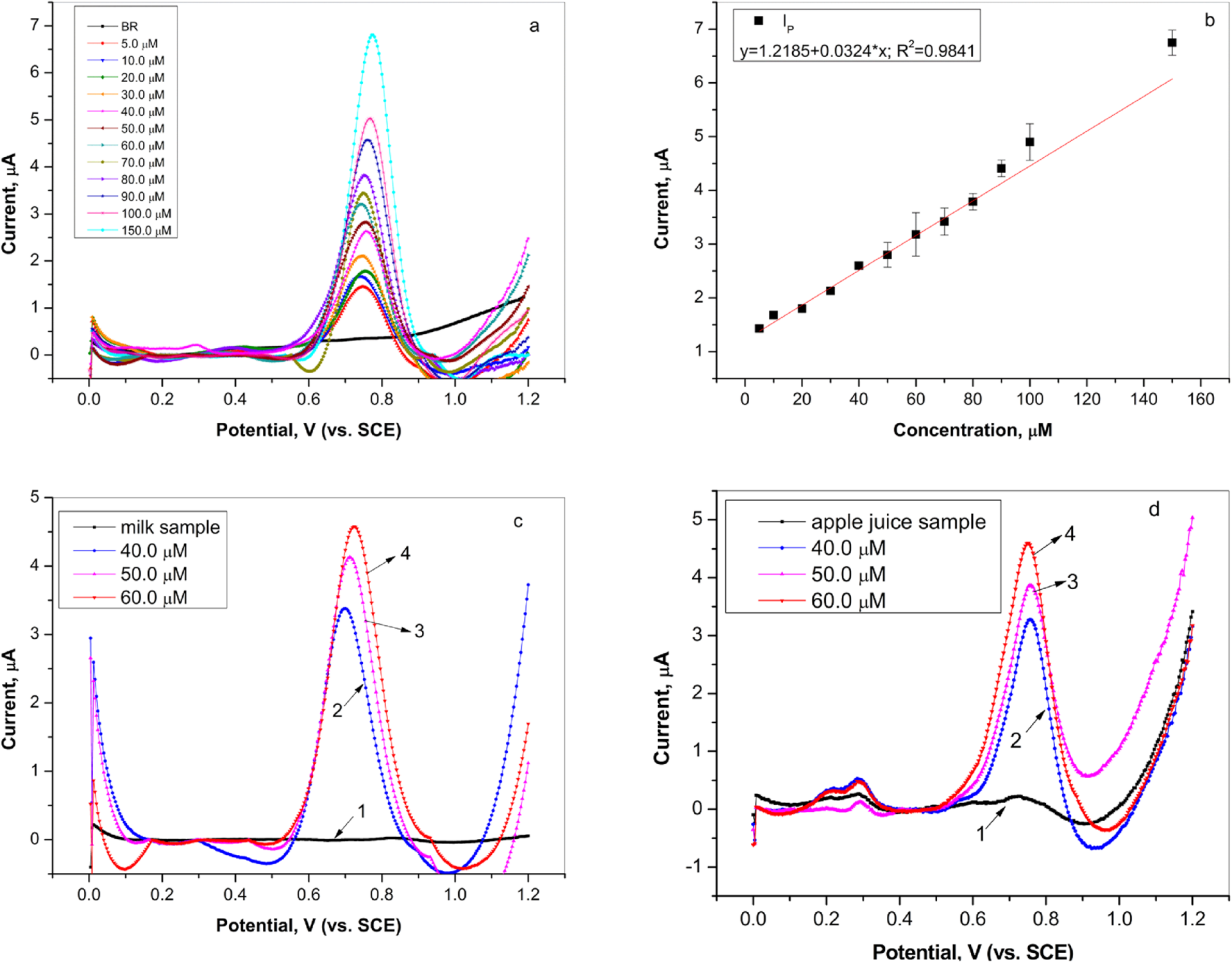
(**a**) Differential pulse voltammogram of L-tryptophan in the concentration range from 5.0 to 150.0 µM on graphite electrode in Britton–Robinson buffer solution (pH 4.0), scan rate 50.0 mV/s (curves subjected to baseline correction); (**b**) The dependence of peak current on L-tryptophan concentration (investigated concentration range 5.0 µM–150.0 µM); (**c**) Differential pulse voltammogram of spiked L-tryptophan in milk sample on graphite electrode: (1) milk sample in Britton–Robinson buffer solution; (2) milk sample with added 40.0 µM of L-tryptophan; (3) milk sample with added 50.0 µM of L-tryptophan, and (4) milk sample with added 60.0 µM of L-tryptophan, scan rate 50 mV/s (curves subjected to baseline correction); (**d**) Differential pulse voltammogram of spiked L-tryptophan in apple juice sample on graphite electrode: (1) apple juice sample in Britton–Robinson buffer solution; (2) apple juice sample with added 40.0 µM of L-tryptophan; (3) apple juice sample with added 50.0 µM of L-tryptophan, and (4) apple juice sample with added 60.0 µM of L-tryptophan, scan rate 50 mV/s (curves subjected to baseline correction).

Differential pulse voltammetry was performed in order to obtain the calibration curve for L-tryptophan determination. Figure 5a represents the constructed differential pulse voltammogram while Fig. 5b shows calibration curve. DPV revealed that the current peak increased linearly with increasing the concentration of L-tryptophan. The corresponding equation, in the examined range of L-tryptophan concentration (5.0–150.0 µM), could be expressed as follows:

5 $$I_{p} \;(\upmu {\text{A}}) = 1.2185 + 0.0324c\;(\upmu {\text{M}});\;R^{2} = 0.9841$$

Further, limit of detection (LOD) and quantification (LOQ) were calculated according to the Eqs. (6) and (7)^31^:

6 $$LOD = 3s{/}m$$

7 $$LOQ = 10s{/}m$$

where s is the standard deviation of peak currents, while m is the slope of the calibration curve. The calculated values of LOD and LOQ were 1.73 µM and 5.78 µM, respectively. The slight variation of the values of peak potential determined in the presence of different concentrations of L-tryptophan was noticed. The reason for such behavior could be explained by the complexity of the electrooxidation mechanism^32^. According to the literature^29,33,34^, the first step, that is, as already mentioned, irreversible two-electron/two proton reaction, was followed by subsequent reactions including several intermediates and products. Besides, the products of L-tryptophan oxidation also had a tendency to get adsorbed on the electrode surface, causing the interference in the electrochemical measurements signals^29,35^. The combination of complex electrooxidation reaction mechanism and adsorption on electrode surface was expressed through the change of peak potential with tryptophan concentration.

To determine whether the graphite electrode was suitable for L-tryptophan detection in real samples, differential pulse voltammetry measurements were performed in milk and apple juice samples (Fig. 5c, d). The milk and apple juice were bought from the local market. Both real samples were prepared by dilution 10-times in Britton–Robinson buffer (pH 4). After that, different concentrations of L-tryptophan (Fig. 5c, d curves 2–4–40.0 µM, 50.0 µM, 60.0 µM) were added to previously prepared milk, as well as apple juice sample and DPV measurements were conducted. The achieved results are summarized in Table 1. The used graphite electrode showed good recovery in the range of 99.3–100.2% with 4.9–8.6% relative standard deviation (RSD) for milk sample and in the range of 99.7–100.2% with 1.4–3.2% RSD for apple juice. The obtained results showed that graphite electrode was suitable for the analysis of L-tryptophan in various real samples.

**Table 1:** Determination of L-tryptophan in milk and apple juice samples in Britton–Robinson buffer solution at graphite electrode (N = 3).

Sample	Added TRP (µM)	Found TRP (µM)	Recovery (%)	RSD
2 (milk)	40.0	39.7 ± 0.68	99.4	4.9
3 (milk)	50.0	50.6 ± 0.47	101.2	8.6
4 (milk)	60.0	59.6 ± 0.43	99.3	5.1
2 (apple juice)	40.0	40.1 ± 0.25	100.2	1.4
3 (apple juice)	50.0	49.7 ± 0.38	99.7	2.2
4 (apple juice)	60.0	60.1 ± 0.21	100.1	3.2

By comparing the values of LOD shown in Table 2, it can be said that the graphite electrode could be used to determine L-tryptophan. Also, it should be noted that an unmodified graphite electrode showed satisfactory results that were comparable to the literature data. In this way, it was shown that recycling zinc-carbon batteries could provide a suitable electrochemical sensor, which was the aim of this research. In further research, the modification of this electrode should be examined in order to improve its characteristics.

**Table 2:** Comparison of the results of previous electrochemical studies for the determination of L-tryptophan.

Electrode	LOD (µM)	Linear range (µM)	Method	References
^a^CuCoHCF/graphite electrode	6.0	10.0–900.0	^b^AMP	^36^
^c^Nafion/TiO_2_-GR/GCE	0.7	5.0–140.0	^d^DPV	^37^
^e^CILE/GNP	4.0	5.0–900	^f^SWE	^38^
^g^MWNTs/MGF/GCE	0.87	5.0–30.0 60.0–500	^d^DPV	^39^
^h^NiCoO_2_/C modifed GCE	5.7	0.0–390.63 390.63–943.4	^d^DPV	^40^
Graphite electrode	1.73	5.0–150.0	^d^DPV	This investigation

^a^Copper-cobalt hexacyanoferrate film modified graphite electrode; ^b^Amperometry; ^c^Glassy carbon electrode modified with a Nafion/TiO_2_-graphene composite film; ^d^Differential pulse voltammetry; ^e^Carbon ionic liquid electrode (CILE) modified with gold nanoparticle (GNP); ^f^Square wave voltammetry; ^g^A multi-walled carbon nanotubes (MWNTs) bridged mesocellular graphene foam (MGF) nanocomposite(MWNTs/MGF) modified glassy carbon electrode; ^h^Carbon-supported NiCoO_2_ nanoparticles (NiCoO_2_/C) modified glassy carbon electrode (GCE).

### Electrode stability and repeatability

**Figure 6:**
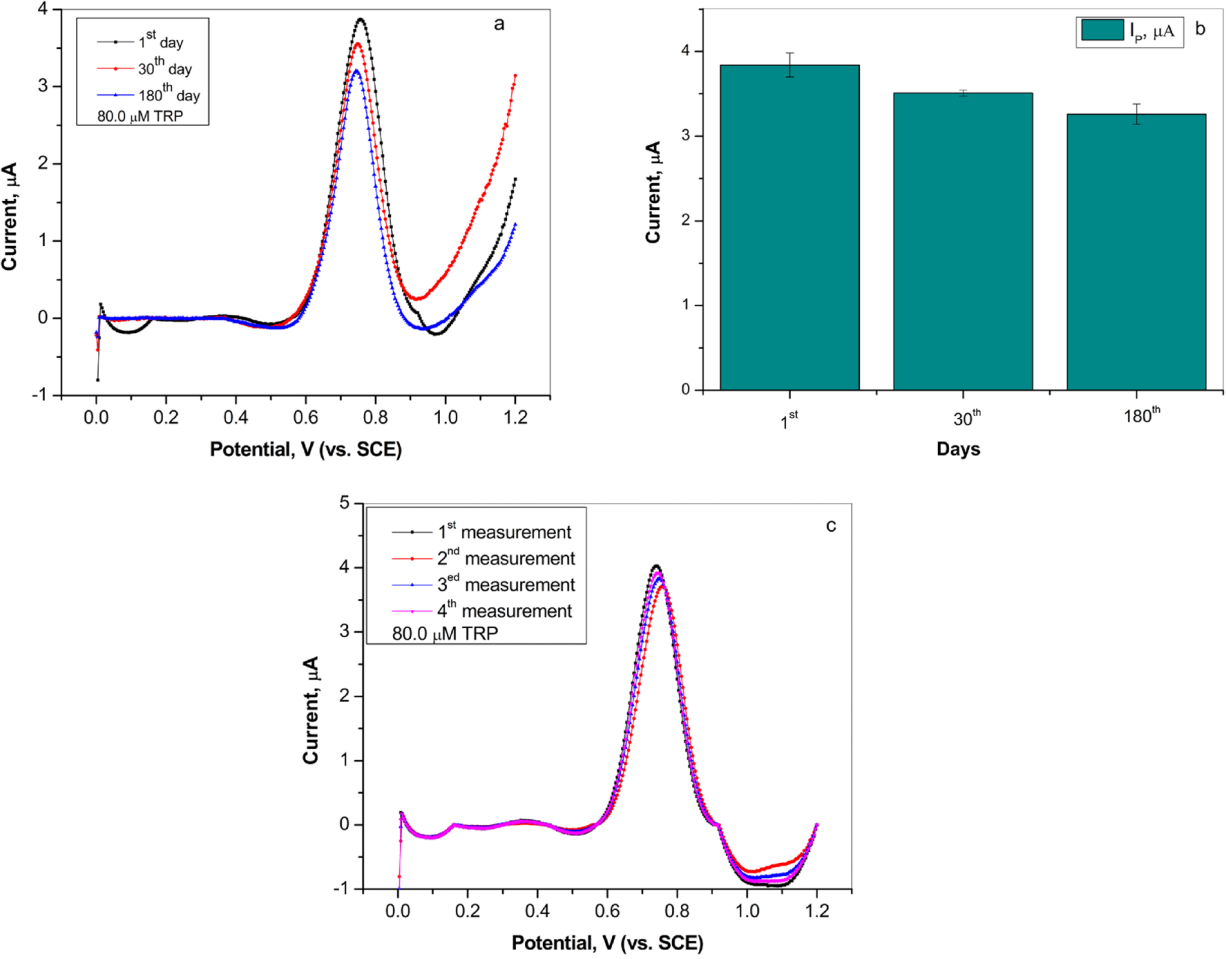
(**a**) Differential pulse volatmmogram for the stability of graphite electrode after 30 and 180 days in Britton–Robinson buffer solution (pH 4.0) containing 80.0 µM L-tryptophan (curves subjected to baseline correction); (**b**) bar diagram for the stability of graphite electrode (**c**) differential pulse volatmmogram for the repeatability (four consecutive measurements) of graphite electrode in Britton–Robinson buffer solution (pH 4.0) containing 80.0 µM L-tryptophan (curves subjected to baseline correction).

In order to examine the stability of the graphite electrode, the experiments were repeated after 30 and 180 days on the same electrode by DPV in Britton–Robinson buffer containing L-tryptophan (80.0 µM) (Fig. 6a, b). According to the bar diagram shown in Fig. 6b, the sensor had good stability because the current peak maintained 91.2% and RSD was 2.9% after 30 days while after 180 days RSD was 3.7% and current peak maintained 84.6%. Further, the repeatability of the graphite sensor was tested using four repeated measurements in a solution containing 80.0 µM TRP and the obtained diagram is presented in Fig. 6c. The calculated RSD 3.0% indicated good repeatability of the graphite electrode.

### Interference study

**Figure 7:**
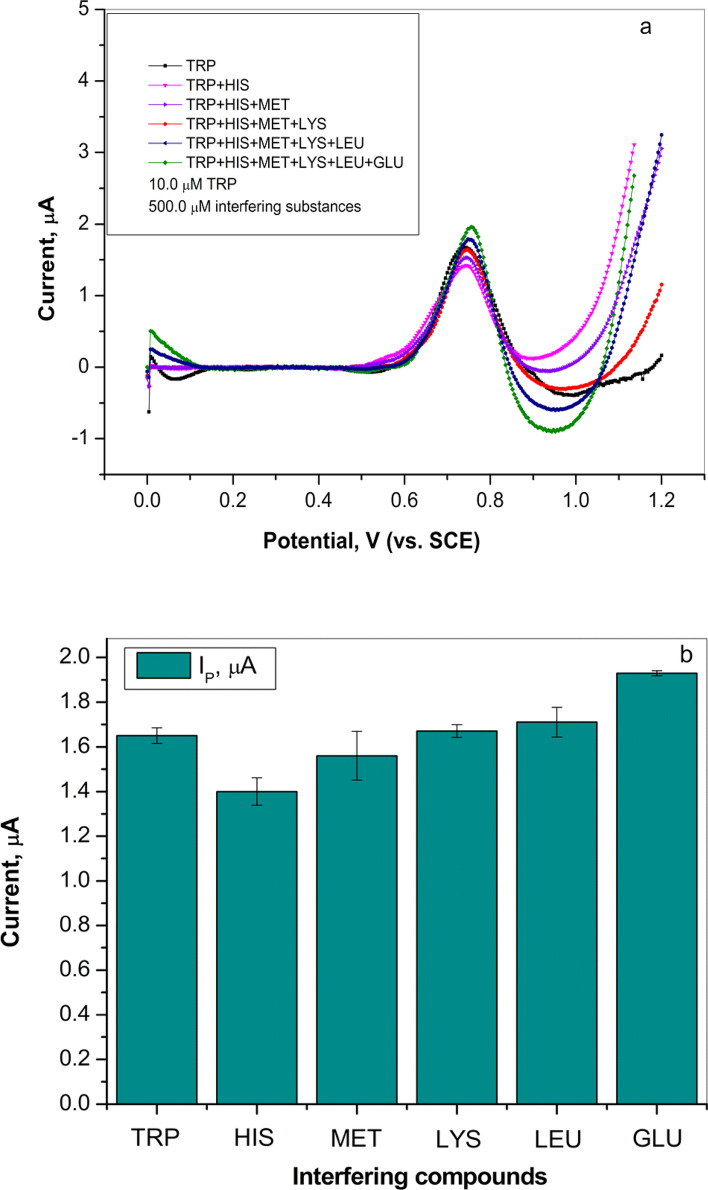
(**a**) Differential pulse voltammetry curves of L-tryptophan (10.0 µM) oxidation in presence of potentially interfering substances (500.0 µM) in Britton–Robinson buffer (pH 4.0) (curves subjected to baseline correction); (**b**) bar diagram between current peak of L-tryptophan in relation to potentially interfering substances.

Investigation of possible interferences for the determination of TRP on the graphite electrode was performed in the presence of other amino acids. Five amino acids including L-histidine (HIS), L-methionine (MET), L-lysine (LIS), L-leucine (LEU) and L-glutamine (GLU) (50-fold content) were chosen for this study. Figure 7a illustrates differential pulse voltammetry curves for 10.0 µM TRP in the presence of the aforementioned amino acids at a concentration of 500.0 µM. L-histidine and L-methionine reduce the current peak, while L-lysine, L-leucine and L-glutamine have positive interference (increase the current peak). According to the graphs shown in Fig. 7a, b, there were no significant changes in the TRP current peak in the presence of these interfering substances. Based on the results, the graphite electrode showed good selectivity for L-tryptophan determination.

## Methods

Pure L-tryptophan, L-histidine, L-methionine, L-lysine, L-leucine and L-glutamine were purchased from Sigma Aldrich (Germany). Britton–Robinson buffer solution was prepared using acetic acid (Zorka Šabac, Serbia), boric acid (Zorka Šabac, Serbia), and phosphoric acid (Merck, North Macedonia)^26^. These used compounds were of analytical purity. NaOH solution was used to adjust the pH of the Britton–Robinson solution. The pH measurements were performed using a pH meter (Eutech instruments). All experiments were performed at room temperature. Electrochemical measurements were done using potentiostats (IVIUM XRE, IVIUM Technologies) with appropriate software in a three-electrode system. Prepared graphite was used as the working electrode, saturated calomel electrode (SCE) and platinum wire were used as reference and auxiliary electrodes, respectively.

The zinc-carbon battery was used as the source of graphite rod. The obtained graphite rod was then prepared by cleaning and drying to remove moisture^21,22^. Further, it was polished by a metallographic process that included grinding on silicon carbide paper, rinsing with water and alcohol, and finally polishing with alumina paste (0.3 μm Al_2_O_3_, Buehler USA). The polished sample was adhered to copper wire using silver glue and then sealed with a methyl methacrylate-based material. Before each measurement, the electrode surface was mechanically polished with silicone carbide paper and alumina paste, then rinsed with distilled water and dried.

Two voltammetric methods, cyclic voltammetry and differential pulse voltammetry, were used in the investigation. Differential pulse voltammetry was conducted under the following conditions: pulse amplitude 50 mV, pulse time 50 ms, within the potential range 0.0–1.2 V (vs. SCE), while cyclic voltammetry measurements were performed from 0 V (vs. SCE) to 1.2 V (vs. SCE). 1000.0 µM of L-tryptophan was utilized as stock solution.

## Conclusion

In this research, the possibility of reusing graphite from waste batteries to prepare an electrochemical sensor was presented. The possibility of utilizing such a sensor was tested in BR buffer in the presence of L-tryptophan, using electrochemical methods, differential pulse voltammetry and cyclic voltammetry. Based on the experimental data, the graphite electrode had sensitivity characteristics for L-tryptophan detection. The cyclic voltammetry indicated an irreversible electrochemical oxidation reaction of L-tryptophan in which the same number of protons and electrons participated. According to the differential pulse voltammetry, the prepared graphite electrode showed significant sensitivity for the determination of L-tryptophan in milk and apple juice samples. Electrode also exhibited good selectivity when several other amino acids were present in the system. Although researchers are increasingly focusing on modifying the surfaces of graphite electrodes, this research has shown that the limit of detection in the µM can also be achieved by using a low-cost material such as graphite rod from batteries.Thus, the tested unmodified graphite electrode showed satisfactory results comparable to the literature data. This electrochemical sensor stands out for its price, ease of operation and can be prepared in a short time. Moreover, the possibility of surface modification allows the characteristics of this sensor to be improved for further analysis in complex media.

## Data Availability

In accordance with the institution's policy, data are not available.
